# Intensity matters: protocol for a randomized controlled trial exercise intervention for individuals with chronic stroke

**DOI:** 10.1186/s13063-022-06359-w

**Published:** 2022-05-24

**Authors:** Lynden Rodrigues, Kevin Moncion, Janice J. Eng, Kenneth S. Noguchi, Elise Wiley, Bernat de Las Heras, Shane N. Sweet, Joyce Fung, Marilyn MacKay-Lyons, Aimee J. Nelson, Diogo Medeiros, Jennifer Crozier, Alexander Thiel, Ada Tang, Marc Roig

**Affiliations:** 1 School of Physical and Occupational Therapy, McGill University, 3654 Promenade Sir-William-Osler, Québec Montréal, Canada; 2 Memory and Motor Rehabilitation Laboratory (MEMORY-LAB), Montréal, Québec Canada; 3 Feil/Oberfeld/CRIR Research Centre, Jewish Rehabilitation Hospital site of CISSS-Laval, Laval, Québec Canada; 4 School of Rehabilitation Science, McMaster University, Hamilton, Ontario Canada; 5 Department of Physical Therapy, University of British Columbia, Vancouver, British Columbia Canada; 6 Department of Kinesiology and Physical Education, McGill University, Montréal, Québec Canada; 7 School of Physiotherapy, Dalhousie University, Halifax, Nova Scotia Canada; 8 Department of Kinesiology, McMaster University, Hamilton, Ontario Canada; 9 Department of Neurology & Neurosurgery, McGill University, Montréal, Québec Canada

**Keywords:** Stroke, Neuroplasticity, Cardiovascular health, Exercise, Clinical trial

## Abstract

**Rationale:**

Cardiovascular exercise is an effective method to improve cardiovascular health outcomes, but also promote neuroplasticity during stroke recovery. Moderate-intensity continuous cardiovascular training (MICT) is an integral part of stroke rehabilitation, yet it may remain a challenge to exercise at sufficiently high intensities to produce beneficial adaptations to neuroplasticity. High-intensity interval training (HIIT) could provide a viable alternative to achieve higher intensities of exercise by using shorter bouts of intense exercise interspersed with periods of recovery.

**Methods and design:**

This is a two-arm, parallel-group multi-site RCT conducted at the Jewish Rehabilitation Hospital (Laval, Québec, Canada) and McMaster University (Hamilton, Ontario, Canada). Eighty participants with chronic stroke will be recruited at both sites and will be randomly allocated into a HIIT or MICT individualized exercise program on a recumbent stepper, 3 days per week for 12 weeks. Outcomes will be assessed at baseline, at 12 weeks post-intervention, and at an 8-week follow-up.

**Outcomes:**

The primary outcome is corticospinal excitability, a neuroplasticity marker in brain motor networks, assessed with transcranial magnetic stimulation (TMS). We will also examine additional markers of neuroplasticity, measures of cardiovascular health, motor function, and psychosocial responses to training.

**Discussion:**

This trial will contribute novel insights into the effectiveness of HIIT to promote neuroplasticity in individuals with chronic stroke.

**Trial registration:**

ClinicalTrials.govNCT03614585. Registered on 3 August 2018

**Supplementary Information:**

The online version contains supplementary material available at 10.1186/s13063-022-06359-w.

## Background and rationale

Exercise training is a recommended core component of comprehensive stroke rehabilitation programs [1, 2]. In the context of stroke, exercise is typically employed on the premise that the brain has a plastic capability (neuroplastic response) to repair itself from neurological damage [3]. A cost-effective method to promote this neuroplasticity is the use of cardiovascular exercise [4]. One bout of exercise performed alone or in concert with non-invasive brain stimulation (e.g., transcranial magnetic stimulation, TMS), may elicit neuroplastic responses that ameliorate motor learning, as studies have demonstrated in healthy individuals [5–7]. Studies in young adults, for instance, demonstrate that cardiovascular exercise can stimulate changes in the excitability of the primary motor cortex (M1) [5–7]. M1 is an important area to target for the promotion of motor function recovery post-stroke [8].

Studies on the acute response to cardiovascular exercise on neuroplasticity in healthy individuals demonstrate that the intensity of the exercise is an integral component in the improvement of neuroplasticity. A single bout of high-intensity interval training (HIIT), a cardiovascular exercise modality that utilizes short periods of high intensities, with interspersed periods of active recovery or rest during the exercise, has demonstrated effectiveness in stimulating neuroplasticity [9, 10] and improving motor learning [11]. Mang et al. reported that 20 min of HIIT at 90% of peak oxygen uptake (VO_2_peak) increased the response to a brain stimulation protocol, amplifying M1 excitability [12]. In contrast, a moderate-intensity continuous (MICT) training protocol (performed at 60% of VO_2_peak) resulted in no benefit to M1 excitability [13].

For individuals with stroke, cardiovascular exercise might also elicit improvement in neuroplasticity and motor recovery [14]. Experiments with animals that have been subjected to a brain lesion have shown that cardiovascular exercise reduces the size of the lesion and inflammation and oxidative stress in perilesional areas [14, 15]. In healthy human subjects, a single bout of HIIT has been demonstrated to be more effective than MICT in stimulating the release of peripheral brain-derived neurotrophic factor (BDNF) [16]. BDNF is a protein that is essential for the promotion of neuroplasticity and motor recovery post-stroke [17, 18]. In young healthy individuals, a single bout of HIIT performed after the practicing of a motor skill can increase BDNF levels, which correlate positively with skill retention, even several days after motor practice [10]. In the context of chronic stroke, acute high-intensity exercise has been described to be associated with larger responses in increased serum BDNF, and additionally greater increases in corticospinal excitability [19, 20].

The importance of higher intensity of exercise is supported by studies which demonstrate the greater improvements in motor learning when motor practice is followed or preceded by a single bout of HIIT, compared to MICT [21]. For instance, studies in individuals with chronic stroke have described that a single bout of MICT may have no effect on M1 excitability, while HIIT can potentially increase affected side excitability, reduce interhemispheric imbalance in excitability, increase neurotrophic factors like BDNF, and improve motor learning [19, 21, 22]. These studies describe a promising methodology of utilizing acute bouts of HIIT exercise; however, eliciting adaptations in brain plasticity that lead to long-term functional recovery (that include functional motor recovery) would require multiple bouts of exercise utilizing a long-term exercise regimen. Therefore, it would be imperative to determine whether multiple bouts of HIIT, in comparison to a MICT program, may result in greater and sustained benefits to neuroplasticity and resultant post-stroke recovery.

### Cardiovascular exercise can improve cardiovascular health for stroke survivors

The most important preventative measure to reduce the risk of stroke is by maintaining ideal cardiovascular health [2, 23, 24]. However, among stroke survivors, cardiovascular comorbidities are highly prevalent. For example, heart disease and hypertension are present in almost 75% and 84% of this population, respectively [25, 26]. Exercise interventions that utilize cardiovascular exercise have been demonstrated to improve cardiovascular risk factors in individuals with stroke such as blood pressure (BP), an important risk factor for primary and secondary stroke [27, 28]. The challenge, however, is that individuals with stroke may be highly sedentary and likely not engage in activities or sustain levels of activity that reduce cardiovascular risk.

Our research group has demonstrated the use of conventional MICT is a safe and feasible method to improve mobility and cardiovascular fitness after stroke [29, 30]. However, like neuroplasticity and motor learning, the intensity of the exercise is a critical component for improving upon cardiovascular health. Our research group has reported greater benefit to cardiac function after 6 months of MICT, when compared to low-intensity exercise [31]. Also observed were improvements in resting BP, among other risk factors, which are only attained when exercise is performed at higher intensities [32].

In order to attain higher intensities of exercise and subsequent benefit to cardiovascular health, HIIT could be a more efficient method than MICT, despite lower amounts of exercise volume. In young adults, for example, 6–12 weeks of HIIT, consisting of short (10–20 min) sessions, performed 3 times per week has been reported to be more effective in improving arterial stiffness, a measure of myocardial demand and coronary perfusion associated with the occurrence of stroke and other cardiac events [33, 34]. A previous meta-analysis has demonstrated that HIIT has resulted in almost a twofold improvement of VO_2_peak compared to MICT [35]. Indeed, HIIT has also been reported to be as or even more effective than MICT in reducing BP [36], for individuals with high BP, despite 20–30% less time devoted to exercise. HIIT could help individuals achieve high intensities that could result in the benefits to cardiovascular health in people post-stroke, which MICT cannot provide. However, a long-term comparison of HIIT vs. MICT for post-stroke individuals has yet to be conducted in a controlled exercise intervention study, in order to fully appraise the extent to which HIIT can yield greater benefit.

### Psychosocial responses to high-intensity exercise are unknown in stroke rehabilitation

While HIIT may provide significant benefits for those with chronic stroke, the adherence to such a physical activity regimen like a HIIT exercise program will depend on several factors such as lack of time [37] and psychosocial determinants such as level of enjoyment [38] and motivation [39]. HIIT may be able to address the prior, as the shorter time commitment has been reported, in healthy populations, as a factor in preference for HIIT in comparison to MICT [40]. Determinants such as enjoyment have been described to be at higher levels in response to HIIT, or similar in comparison to MICT [40–42].

Considering that in the stroke population, the intensities that are achieved may be psychologically demanding, due to heightened cardiovascular and neuromotor effort, it is surprising that an examination of psychosocial factors in HIIT or MICT training for the stroke population is not currently available. An understanding of psychosocial indicators of exercise, like enjoyment and motivation, and how HIIT may affect such outcomes, would provide important information about the potential sustainability of HIIT and its applicability in stroke rehabilitation.

### Objectives

The primary objective of this study is to compare the effects of 12 weeks of HIIT vs. MICT on neuroplasticity. Changes in neuroplasticity will be determined by examining different markers of excitability in upper limb representational areas of M1 with TMS. In addition, the secondary objectives of this study are to compare the effects of HIIT and MICT on measures of cardiovascular health, motor function, and psychosocial responses to exercise. Change in cardiovascular health will be measured by assessing resting BP, arterial stiffness, cardiorespiratory fitness (VO_2_peak), and waist-hip ratio. Psychosocial responses to exercise will examine the effect of HIIT vs. MICT on motivation and enjoyment in response to the respective exercise intervention. Motor function parameters of gait speed, walking capacity, and upper limb motor learning will also be assessed. The exercise interventions of HIIT and MICT will be conducted using a whole-body paradigm, utilizing recumbent steppers.

### Hypotheses

We hypothesize that HIIT will be more effective in promoting neuroplasticity. HIIT will increase the excitability of upper limb representational areas on M1 of the lesioned hemisphere. This will lead to a reduction in the imbalances in M1 excitability that is typically observed in chronic stroke [43, 44]. Based on previous findings [22], we will also expect that the increases in M1 excitability in the lesioned hemisphere after HIIT will be partially mediated by a reduction in intracortical inhibition [45], which is a marker that has been described to increase post-stroke, negatively affecting recovery [8]. HIIT is also expected to be more effective in improving cardiovascular measures of resting BP, arterial stiffness, and VO_2_peak [32, 35]. We predict that after a period of 8 weeks post-termination of the training program (T2), these improvements in neuroplasticity and cardiovascular health will be maintained after to a greater extent in the HIIT group, in comparison to the participants in the MICT group.

We also anticipate greater improvement in walking speed [46], and motor learning post-HIIT, due to an increase in neuromuscular recruitment due to high-intensity exercise, and the aforementioned changes in neuroplasticity, respectively [22]. In contrast, we will also expect a similar improvement in walking capacity in response to either HIIT or MICT. Additionally, responses of motivation and enjoyment will be similar in both groups.

## Methods

The following sections describe the current study protocol (version 1.0 — Dec 2021). This study protocol is presented in accordance with the Standard Protocol Items: Recommendations for Interventional Trials (SPIRIT) guidelines [47].

### Study design

**Fig. 1:**
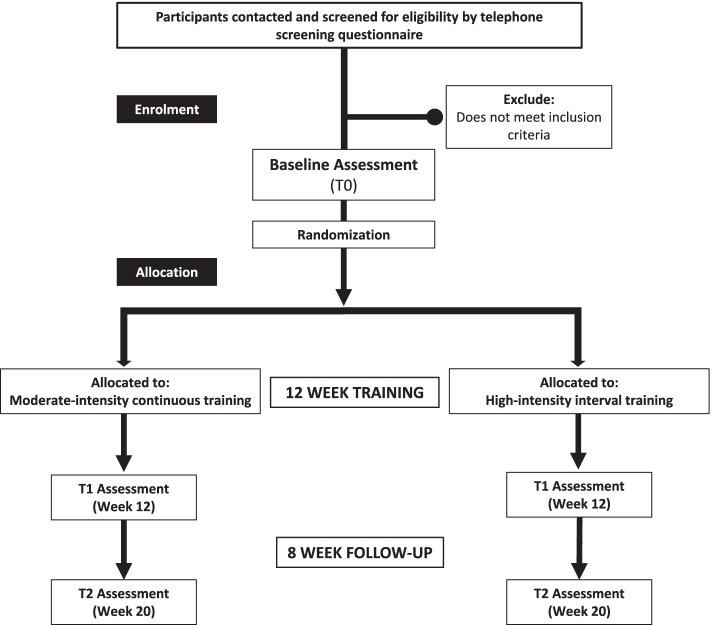
Study flow diagram

This study is a two-arm, parallel-group multi-site RCT (Additional file 1). Outcomes will be assessed at baseline (T0, week 0), at the end of the intervention period (T1, week 12), and at 8-week follow-up (T2, week 20) (Fig. 1). T2 will allow us to evaluate the long-term effects of HIIT and MICT. Assessors will be blinded to group assignment prior to completion of T0. Due to limitations in staffing and resources, assessors and exercise instructors will not be blinded to group assignment.

### Sites

This study will be conducted at the Jewish Rehabilitation Hospital (Laval, Québec, Canada) and McMaster University (Hamilton, Ontario, Canada).

### Participant eligibility

Participants will be recruited from the Jewish Rehabilitation Hospital (JRH) and Ontario Central South Stroke Network (Hamilton, ON). We will recruit individuals who are in the chronic phase of stroke, which will allow us to test whether HIIT can promote neuroplasticity at later stages in recovery, where changes in neuroplasticity are potentially more difficult to achieve [3]. By recruiting individuals in the chronic stage of stroke recovery, we will also reduce the inter-individual differences in brain excitability, that are observable in the subacute stage of stroke recovery. Participants will be contacted by telephone and screened for eligibility via a screening questionnaire addressing the following inclusion/exclusion criteria:

#### Inclusion criteria

Inclusion criteria include 40–80 years old; 6–60 months following first-ever, single stroke confirmed by MRI/CT; living in the community and able to independently ambulate at least 10 m (the use of assistive devices is permitted, as many individuals who do regain some ability to walk following stroke do so with some adaptation); and the ability to follow instructions in exercise and assessments. If individuals have communicative difficulty due to speech or language deficits (e.g., aphasia), admission into the study is to be done on a case-by-case basis based on judgment taken by the research group.

#### Exclusion criteria

Exclusion criteria include significant disability, as determined by a modified Rankin scale score of > 2; actively engaged in stroke rehabilitation services or a structured exercise program in addition to the one provided by this study; class C or D American Heart Association Risk Criteria [48]; other neurological or musculoskeletal comorbidities that will prevent safe exercise participation; pain that is made worse with exercise; cognitive, communication, or behavioral issues that may limit safe participation in the exercise program; and contraindications to TMS [49].

Informed consent to participate in the study, prior to entry, will be obtained from the participant by study coordinators, LR and KM, at JRH and McMaster sites, respectively.

### Recruitment strategy

At the JRH site, participants will be admitted from clinician referrals and access to participant databanks from patients who have consented to be contacted for participation in research projects. The JRH stroke program admits approximately 190 patients per year and currently conducts a cardiovascular exercise program that will allow us to recruit individuals who have the most interest in exercise and will meet the inclusion criteria of our stroke study, like those conducted previously in at this site [22].

At the McMaster site, the Ontario Central South Regional Stroke Network and the Regional Rehabilitation Centre at Hamilton Health Science can provide access to the potential 600 new patients with stroke admitted per year. The Regional Rehabilitation Centre will serve as the primary source of recruitment for this site. The McMaster site has already utilized successful strategies for the recruitment of such participants for large community-based exercise trials through these networks.

### Randomization

The unit of randomization will be the participant. The randomization process will use a computer-generated group assignment (http://www.randomizer.org) with a 1:1 allocation ratio into either HIIT or MICT training groups. Participants will be stratified between both sites (JRH and McMaster), and we will conduct the randomization with a variable block size unknown to each site. PIs MR and AT will conduct and conceal the allocation of their counterpart’s site (MR for McMaster, AT for JRH). Upon obtainment of participant consent and completion of baseline assessments (T0), group allocation will be revealed to the participant and assessor.

### Interventions

All interventions will take place at the JRH and McMaster site facilities. HIIT and MICT will consist of 12 weeks of training with three sessions per week performed on alternate days to avoid overtraining and maximize adaptations [50]. Initially, training was to be performed in a group training session on recumbent steppers with a 2:5 trainer-to-participation format, as group training may be an important facilitator for adherence to exercise in stroke [51]. However, to reduce the risk of COVID-19 infection, we  have been using a 1:1 trainer-to-participant format during training sessions. Recumbent steppers were chosen because they will; 1) allow participants with a wide range of functional abilities to exercise at high intensities [52]; 2) have been previously described as safe and effective for implementing HIIT [53] and MICT [54] training protocols in stroke populations and allow for exercise intensity to be controlled in an easier manner than using treadmill training; 3) reduce the risk of falls due to the seated position required during exercise; and 4) involve both upper and lower limbs, training muscles that are evaluated in TMS and motor function outcomes, facilitating an examination of relationships between upper and lower limb functions. Heart rate (HR), rate of perceived exertion (RPE), and, if needed, BP will be monitored continuously throughout each training session as individuals who take beta-blockers will exhibit a blunted HR response.

Exercise intensity will be determined using the HR reserve (HRR) method calculated as HRR = (max HR at peak VO_2_peak – resting HR) × (% exercise intensity) + (resting HR), in combination with RPE [55]. For participants taking HR limiting medication (e.g., beta-blockers), a modified HRR equation will be used (HRR = 0.8 × [max HR at peak VO_2_peak – resting HR] + [resting HR]) [56]. Both MICT and HIIT will involve 3-min warm-up and 2-min cool-down periods at 30% of HRR.

#### HIIT

**Fig. 2:**
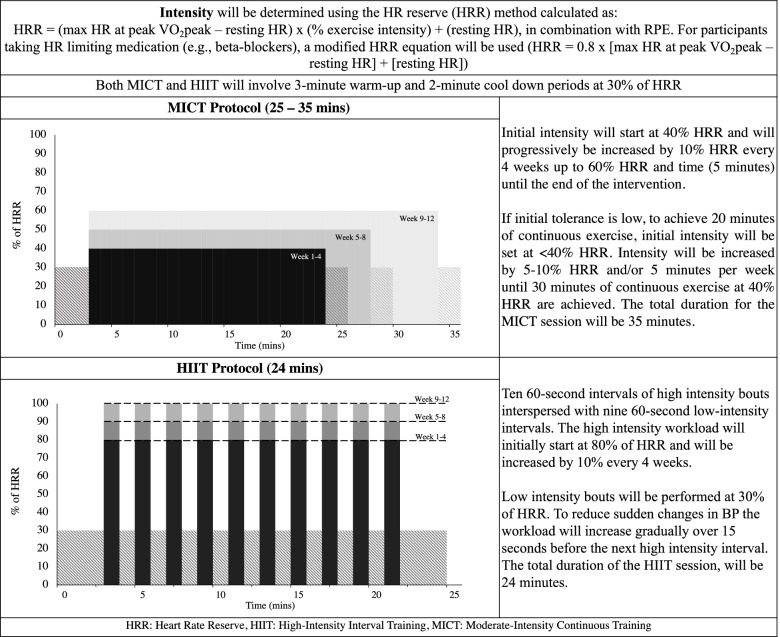
MICT and HIIT protocol

We will use an adjusted HIIT protocol that has been described previously in individuals with chronic stroke [57]. It will involve ten 60-s intervals of high-intensity bouts interspersed with nine 60-s low-intensity intervals [57] (Fig. 2). The high-intensity workload will initially start at 80% of HRR and will be increased by 10% every 4 weeks. Low-intensity bouts will be performed at 30% of HRR. To reduce sudden changes in BP and ensure that target intensity is achieved [58], the workload at the low-intensity interval will increase gradually over 15 s prior to the next high-intensity interval. The total duration of the HIIT session will be 24 min.

#### MICT

We will use a conventional MICT protocol typically employed in stroke rehabilitation programs. Initial intensity will start at 40% HRR and will progressively be increased by 10% HRR every 4 weeks up to 60% HRR and time (5 min) until the end of the intervention (Fig. 2). If initial tolerance is low, to achieve 20 min of continuous exercise, the initial intensity will be set at <40% HRR. Intensity will be increased by 5–10% HRR and/or 5 min per week until 30 min of continuous exercise at 40% HRR are achieved. The total duration for the MICT session will be 35 min.

### Adherence and modification to interventions

Adherence to training in both HIIT and MICT groups will be monitored by exercise trainers who will track attendance in training sessions and adherence to required training intensities as described in this protocol (see below), using the Borg scale and Polar HR devices. If participants miss sessions of training, they will be offered make-up sessions to complete the full 36 sessions of training. During the period between T1 and T2, study coordinators will contact participants by telephone to encourage participant retention at T2. These calls will also serve to assess activity level post-training (see below).

If participants desire to stop training and develop health conditions or injury that preclude safe participation of exercise over the course of the intervention, we will discontinue training. Participants will be asked to not participate in another structured exercise regimen or intervention over the course of their participation in either the HIIT or MICT group; otherwise, they will not be allowed to continue participation. However, during the period between T1 and T2, participants will not be asked to refrain from further participation in exercise. This may influence outcomes at assessments at T2, as participants may engage in another exercise program after completing T1 assessments. In order to address this, we will use the Physical Activity Scale for Individuals with Physical Disabilities (PASIPD) [59] and administer it midway in between T1 and T2.

## Outcomes

### Assessments

**Fig. 3:**
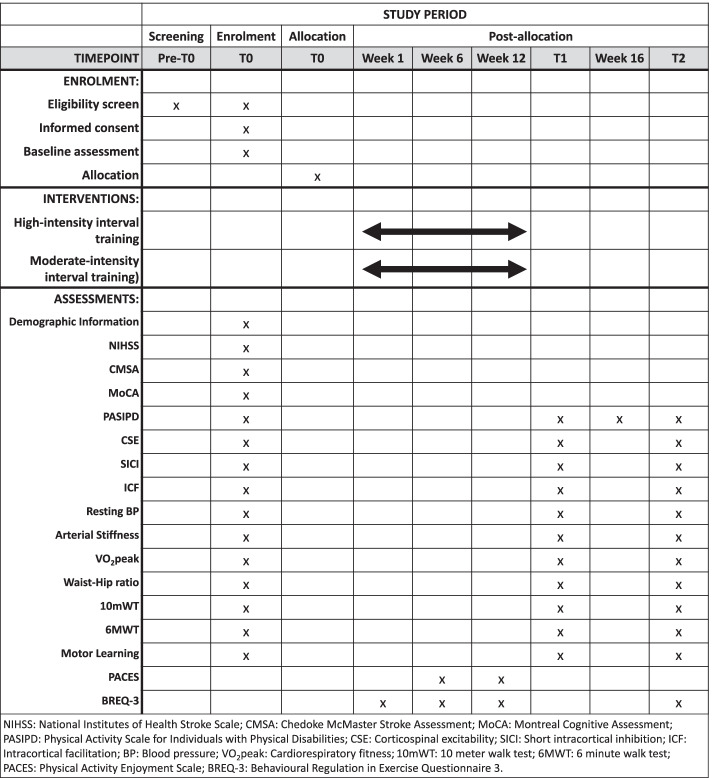
Schedule of enrolment, interventions, and assessments

Prior to the initial assessment, the assessor will obtain written consent from the participant. Baseline descriptive characteristics will be collected at T0 and include biological sex, age, weight, height, body mass index, education level, characteristics of stroke, and past medical history. Additionally, the degree of neurological deficit (National Institutes of Health Stroke Scale, NIHSS [60]), upper limb motor function (Chedoke McMaster Stroke Assessment, CMSA [61]), and global cognitive function (Montreal Cognitive Assessment [62]) will be assessed at baseline. Medication use will be recorded at all time points. PASIPD [59] will be assessed at all time points and midway through the 8-week follow-up (week 16), to account for changes in physical activity between T1 and T2. All outcomes, apart from enjoyment, will be collected T0–T2 at both sites. To minimize participant fatigue, assessments will take place over 3 days (1.5 h each). Data collection forms can be provided by study PIs upon request. Figure 3 provides a timeline and collection of outcomes.

### Primary outcome

#### Neuroplasticity

Our study will assess changes in neuroplasticity with TMS applied to the representational areas of the hand’s first dorsal interosseous muscle (FDI) on M1. We selected this muscle because it can be elicited at relatively low stimulation intensities that are more comfortable for the participant [63] and because it is involved in upper limb activities of daily living such as grasping objects. We will also apply TMS on both hemispheres [21, 22], so that we can assess whether exercise will restore imbalances in interhemispheric excitability, an important marker of motor recovery after stroke [44].

##### General TMS procedures

Electromyography will be recorded from the FDI on the participant’s affected and unaffected sides. The position of electrodes will be photographed (with permission from the participant), to ensure repeatability in all assessments (T0–T2). Using neuronavigation (Brainsight, Rogue Research), we will co-register the heads of participants to a generic MRI template to identify and mark optimal areas for stimulation (a “hotspot”) on M1 to elicit a motor-evoked potential (MEP). The amplitude of the MEP will indicate the level of muscle activation in response to TMS, used to quantify excitability. Neuronavigation software will allow us to save the “hot-spot” of each person in order to repeat the stimulation with better spatial accuracy [64]. Once the “hot-spot” has been identified, we will determine the resting motor threshold (RMT), the minimum intensity to obtain 10 MEPs with an amplitude of 0.05μm out of 20 stimulations [63]. Electrical stimulation over the median nerve (FDI) will be used to assess changes in muscle contractility. This assessment has been demonstrated to be feasible for patients with stroke [65], and our laboratory has shown that normalizing MEP amplitude to muscle contractility is important for obtaining accurate corticospinal excitability data in exercise studies which utilize applied TMS at different time points [9].

##### Primary outcome: corticospinal excitability (CSE)

CSE will be measured using a single pulse TMS protocol. Two blocks of 25 stimulations, per hemisphere, that are elicited at an intensity of 120% of the resting RMT will be delivered over the FDI “hotspot” at rest and during an isometric contraction performed at 10% of the maximal force. This number of stimulations is sufficient to obtain a reliable measure of CSE using TMS [66]. Each stimulation will be applied 5s apart to reduce the effects of repetitive TMS on excitability [67]. A total of 50 stimulations will be averaged to obtain the composite MEP amplitude [9, 68]. MEP amplitude is examined because it can predict exercise-induced improvements in procedural memory and skill retention in healthy subjects [9] and motor recovery in stroke [8].

### Secondary outcomes

#### Neuroplasticity

##### Secondary outcome

Short intracortical inhibition and intracortical facilitation (SICI and ICF)

SICI and ICF will be measured using a paired-pulse TMS protocol that will use a conditioning stimulus of 80% RMT, followed by a supra-threshold stimulus (120% RMT) delivered at rest after 2.5ms (inhibition) and 12ms (facilitation), respectively. These intervals between stimuli capture changes in excitability after motor learning [69] and are susceptible to change with exercise in people with chronic stroke [22]. The amplitude of the MEP elicited by the send stimulus normalized to the MEP amplitude at baseline will provide an estimate of inhibition and facilitation [45]. A total of 25 paired pulses (separated by 2s), per hemisphere, will be delivered at the FDI. Our laboratory has used these measures of excitability in a previous study to investigate the effects of a single bout of HIIT in individuals with chronic stroke [22]. ICF and SICI are important markers of neuroplasticity, specifically related to motor learning processes [45, 70], and have been used to predict motor recovery post-stroke [8].

#### Cardiovascular health

##### Secondary outcome — resting BP

Resting BP will be taken at the brachial artery of the less affected arm using an automated BP monitor. Two readings will be taken and averaged, and if values differ by > 5 mmHg, 2 more readings will be taken, and another average will be taken for 4 readings.

##### Secondary outcome — arterial stiffness

Central pulse wave velocity (PWV) represents the pulse wave propagation within the arterial tree [71] and is considered the gold standard marker of arterial stiffness. PWV will be measured using applanation tonometry, a non-invasive technique, to capture pulse waveforms at the carotid and femoral arteries. Central PWV will be calculated by the following equation: PWV= (meters, *D*)/(seconds, Δ*t*), where Δ*t* is the travel time of the propagated wave between carotid and femoral arteries, and *D* is the distance between the two locations being recorded [72]. An increase in PWV is associated with an increased risk of cardiovascular morbidity and mortality, respectively [73]. A previous study has described an inverse relationship between PWV and physical fitness post-stroke [74].

##### Secondary outcome — cardiorespiratory fitness

Cardiorespiratory fitness will be assessed by assessing the VO_2_peak of participants. VO_2_peak will be determined by a graded cardiopulmonary exercise test (CPET) on a recumbent stepper which has been validated for individuals with stroke [54] and has been used by our lab in a previous study [22]. Cardiovascular responses will be monitored using a 12-lead ECG and BP monitor. RPE will be recorded during the exercise test [75].  Each site will have a medical support team, including an on-call physician, in the case of any medical emergency during this assessment.

##### Secondary outcome – waist-hip ratio

Waist-hip ratio is among the strongest predictors of risk for stroke, independent of other vascular risk factors [76]. It will be determined from the waist circumference measured at the level of the umbilicus, and hip circumference will be taken at the level of the greater trochanters. Markers of abdominal obesity are easily measured in clinical settings and are stronger predictors of cardiovascular events [77, 78] than body mass index.

#### Motor function

##### Secondary outcome — gait speed

We will assess gait speed using a self-paced 10-m walk test (10mWT). Participants will be timed starting at the 4-m mark, up until the 8-m mark of the 10-m course. Speed over 6 m will provide the result for one trial. The average of both trials will be the outcome for gait speed. Participants will be permitted to use gait aids (e.g., walker, cane) during this assessment.

##### Secondary outcome — walking capacity

The 6-min walk test (6MWT) will be used to measure walking capacity. Participants will walk along a 20-m straight course as many times as possible within 6 min [79]. The primary outcome of this test is the distance walked (in meters) during the test. Pre and post-test resting BP, HR, and RPE will also be assessed. During this test, participants will be permitted to use gait aids.

##### Secondary outcome — motor learning

For this study, we will use a computerized visuomotor task that will require modulating force [22] during a hand-grasping, as this skill is essential in the performance of many activities of daily living. The participant will be seated in front of a computer screen and be asked to make a fist applying force with their most affected hand on a handgrip. If the affected hand is unable to hold or modulate force on the handgrip due to disability, the unaffected side will be used.

This handgrip will control the position of the cursor displayed on the screen. The goal of the motor task will be to apply appropriate force in order to move the cursor so that it touches as many targets on the screen, as accurately as possible. Participants will perform 4 blocks of 20 trials, and the score for each block will be calculated as the total time that the cursor is on the target areas, divided by the total time of each trial, multiplied by 100.

#### Psychosocial responses to exercise

##### Secondary outcome — enjoyment

Enjoyment will be assessed after week 6 and T1, using the Physical Activity Enjoyment Scale (PACES), which has been validated for older adults with functional limitations [80] and people with multiple sclerosis [81]. Participants will be asked to respond to the prompt, “Please rate how you feel at this moment about the exercise you have been doing” in relation to several domains using a 7-point scale (e.g., 1=does not make me happy, 7=makes me happy). A total score is calculated (ranging from 8 to 56). Higher scores indicate greater enjoyment.

##### Secondary outcome — motivation

The Behavioral Regulation Exercise Questionnaire-3 [80, 82] will be used to assess participants’ motivation for physical activity at T0, T1, and T2 and will be administered after the 1st week of training and at the end of training. Participants will respond to 24 items using a 5-point Likert scale (e.g., 0=not true for me, 4=very true for me), covering different types of motivations. Autonomous motivation (i.e., internally regulated motivation) scores will be calculated by averaging the score on the identified, integrated, and intrinsic regulation subscales. External motivation will consist of the mean of the external and introjected regulation scores. Earlier versions of this questionnaire (BREQ-2) have been described as reliable and have been validated questionnaire previously in individuals with coronary heart disease [83, 84]

For both questionnaires, French or English versions will be provided depending on the language preference of the participant. If a participant has a speech or language difficulty, assessors will utilize a third party to provide clarification of the questionnaire items and participant responses.

### Sample size estimation

Based on previous data [21] and using a linear mixed model, we estimated that we would require 32 participants per group (*n* = 64 total) to detect a 5% increase at T1 in the resting MEP amplitude and intracortical inhibition of the affected hemisphere’s M1 in the HIIT group. An increase of 5% is associated with improved motor function in stroke [2]; therefore, we will consider this difference between HIIT and MICT groups to be clinically significant. The statistical package G*Power was used to determine the sample size required to obtain a power of 80% (alpha < 0.05). We have increased the sample size to 40 per group (*n*=80 total) to accommodate for a 20% attrition rate.

### Statistical analysis

Estimates of the effect of HIIT and MICT at the end of the intervention (T1) for all outcomes will be determined using linear mixed models. Baseline scores (at T0), age, and sex will be included as covariates for sub-analysis. The primary analysis focuses on T1, but T2 will also be included in the model (except for enjoyment, which will not be measured at T2) to increase the statistical efficiency of the estimate. If group interaction effects are significant, *t*-tests based on the linear mixed models will be employed as planned pairwise comparisons to determine differences between HIIT and MICT. Sex-based subgroup analyses will also be performed for exploratory purposes.

Participant data will be analyzed based on intention-to-treat; therefore, linear mixed models will be the preferred method of analysis as it is more flexible than the analysis of variance, and allows for the correlation of repeated measures within subjects and missing data, as long as missing data are missing at random [85]. Interactions between changes between primary and secondary outcomes will be explored with the Freedman-Schatzkin test, which allows for the identification of mediators of change in small-scale exercise studies [86].

### Blinding

Participants will be blinded to group allocation until the completion of T0 assessments. Due to constraints on resources, assessors will not be blinded to group allocation. However, participants will not be told that there is another training group and will not have sessions in which they train while a participant of another training group also takes place. Group allocation will be blinded in data analysis.

### Data management

Data will be collected from assessments and recorded on paper case report forms. Data from these forms will be entered and stored on a secure database that is shared between both sites. Paper case reports will be stored in a secure location at each site. All information collected from participants will be recorded under a given subject identifier that will be specific to the study site (JRH site: IM201, IM202 … ; McMaster site: e.g., IM101, etc.).

### Confidentiality

Any identifying information, outside of relevant outcome data, will not be provided to the opposite site and will be stored separately from collected outcome data. Requests for data from either site will require the expressed permission of the site’s PI (JRH: MR, McMaster: AT). Access to JRH and McMaster databases will be password protected with only MR and LR, and AT and KM having access to JRH and McMaster databases respectively.

### Safety and adverse event monitoring

Adverse events that are related or unrelated to training, including but not limited to, injuries, falls and muscle soreness, or fatigue, that affect activities of daily living, will be asked about and documented by instructors prior to each training session.

### Protocol modifications

Any changes to the current study protocol (version 1.0) will be communicated to study investigators and the research ethics boards of the Centre de Recherche de Readaptation du Montréal and Hamilton Integrated Research Board and electronically to the trial registry (ClinicalTrials.gov).

### Oversight and monitoring

The steering committee will be comprised of 4 investigators (LR, KM, MR, and AT) and will hold quarterly meetings to monitor trial activity, and be responsible for the dissemination of results (i.e., manuscripts, presentations, and knowledge translation). LR and KM will be responsible for communication among investigators, coordinating meetings or teleconferences, coordinating staffing, and assisting in training workshops and day-to-day trial activities. The External Advisory Committee will include a physician in stroke rehabilitation, physical therapist, healthcare administrator, and a person with stroke. They will ensure that the study design and interpretation of findings are applicable to current practice.

The Data and Safety Monitoring Committee will comprise three individuals external and independent to the study (a statistician, a physician, a physical therapist) to audit study progress, review adverse events, and advise termination of the study if the safety data are of sufficient concern. All adverse events will be immediately reported to this committee, as well as relevant ethics boards.

### Dissemination plans

Collaborators who have actively participated in the concept of the study design, protocol development, and acquisition of data will be invited to co-author subsequent output from this study. Results will be disseminated through peer-reviewed publications and scientific presentations as well as through the investigators’ professional networks (Canadian Partnership for Stroke Recovery, Canadian Stroke Consortium, Ontario Central South Regional Stroke Program activities, Quebec Rehabilitation Research Network) and local stroke recovery groups. Exercise and rehabilitation specialists will be informed during presentations of the results of the study in our affiliated clinical sites across Canada (e.g., JRH, GF Strong Rehabilitation Centre). We will also capitalize on other initiatives led by our research team to facilitate knowledge transfer and exchange that include different stakeholders.

Datasets compiled from the acquisition of participant’s data during this study, and statistical code used to analyze participant data will be made available in the repository of journals upon publication and/or available upon request.

## Discussion

Cardiovascular exercise is a valuable tool in stroke rehabilitation, as it promotes mechanisms of neuroplasticity, and improves upon measures of cardiovascular health, ameliorating recovery and reducing the risk of stroke recurrence. Cardiovascular exercise, in the form of MICT, is already widely employed in stroke exercise rehabilitation, but the challenge that remains is how stroke rehabilitation professionals can implement exercise at intensities that produce the desired clinical effect (i.e., HIIT).

Following this, it is also crucial to understand how higher intensities of exercise can mediate improvement in neuroplasticity and cardiovascular health. Improvement in these domains is vital for functional recovery and reducing the burden of disability in individuals recovering from stroke. Understanding the factors which influence participation and adherence in these types of exercise can help stroke rehabilitation professionals to more effectively implement and tailor exercise prescription during treatment.

This study will be among the first to comprehensively compare the effectiveness of multiple bouts of HIIT and MICT on important determinants of stroke recovery: neuroplasticity, cardiovascular health, and motor function. In addition, this study will also examine the psychosocial response to exercise, which is important for the maintenance of exercise behaviors and participation in training modalities like HIIT. The benefit of exercise for reducing recurrent stroke events and promoting functional recovery is well-known [2]; however, no studies, to our knowledge, have compared the effects of HIIT vs. MICT for simultaneously improving neuroplasticity and cardiovascular health. Furthermore, few studies have examined aspects such as enjoyment and motivation, in this clinical population, which could be important for sustainability. Understanding the long-term effects of such interventions is also lacking in the current literature, and this study will also address outcomes in this context, by including an 8-week follow-up assessment.

This two-arm multi-site study design will allow for an efficient recruitment of individuals to meet the target sample size. Both study sites are established in settings which will provide access to a large population of individuals recovering from stroke. The prescription of exercise, by determining HRR, will be personalized to the capacity of the participant at baseline and will also consider RPE and the use of medications such as beta-blockers, in order to accurately prescribe exercise intensities depending on the group. This will enable participant engagement and adherence to the study protocol, while also safely implementing the exercise program while progressively increasing the intensity of exercise. The apparatus for exercise, NuStep Recumbent steppers, will enable the safe participation of exercise by limiting the risk of falls and injury, in comparison to the treadmill or stationary bike exercise.

In response to COVID-19-related institutional regulations and developed guidelines [87], group training will not be permitted, but this will therefore necessitate a 1:1 instructor-to-participant supervision. As part of the exercise instruction, data collection of heart rate and workload will be more readily collected and reported, and vital signs in response to exercise, such as RPE and BP will be much more easily assessed over the course of training. The collection of this data, particularly the heart rate and workload data, will allow us to examine the potential efficiencies of HIIT and the ability of participants to attain prescribed intensities.

The inclusion of an 8-week follow-up in this study is an important aspect that will allow us to investigate the long-term effects of HIIT and MICT, after culmination of an exercise intervention, which is understudied in the stroke population. By examining all outcomes after an 8-week period, this study will be able to assess the sustainability of benefits after participation in cardiovascular exercise. By examining this in the context of comparing HIIT and MICT, we will be able to determine whether the intensity of exercise mediates the long-term effects on study outcomes.

The design of this study does have several limitations. Firstly, there are limitations in the blinding of subjects and assessors. As mentioned previously, due to a lack of resources, staffing availability has made it not possible to blind assessors and exercise instructors from group assignment. Therefore, we are also unable to blind assessors who have conducted assessments at baseline, T0, and T2.

Secondly, the implementation of HIIT or MICT itself may influence psychosocial responses of enjoyment and motivation. Exercise instructors may require more interaction with participants in the HIIT group, because of the frequent change in intensity level and opportunity for feedback and use of encouragement. This may not occur to the same extent in the MICT group due to the continuous level of intensity. Our research group has implemented several methods, including standardized feedback across both groups to limit these potential effects of HIIT and MICT exercise instruction.

As a multicenter study, this setting may lead to potential inconsistencies between sites with respect to outcome assessments and exercise instruction. This necessitates that there is careful monitoring and standardization of assessment techniques and exercise instruction. Our research group, and our Steering committee in particular, will coordinate training sessions, to implement proper assessment techniques and standardization in both study sites, and regular meetings to maintain adherence to standard operating procedures.

### Impact of the study

As mentioned previously, there is strong evidence for the use of cardiovascular exercise, and it is a recommended component of stroke rehabilitation [1]. As it currently stands, the conventional use of MICT could be an insufficient cardiovascular stimulus to elicit improvement in neuroplasticity and cardiovascular health [88]. Attaining higher intensities of exercise using HIIT could provide stroke rehabilitation professionals an effective method to further improve stroke recovery. This evaluation of HIIT and MICT will provide knowledge on the benefits to multiple domains pertaining the stroke recovery and adherence to exercise behavior. Physical therapists and other clinicians using exercise in stroke rehabilitation will benefit from a greater understanding of the potential efficiency of HIIT as an exercise modality, and as an effective method to attain high intensities of exercise in the stroke population

This study will provide a comprehensive examination of the efficacy of a novel HIIT, in comparison to an established MICT method, for promoting neuroplasticity and cardiovascular health, while also addressing the underlying psychosocial responses to these exercise modalities. An understanding of the difference between HIIT vs. MICT exercise rehabilitation methods will provide a greater understanding into intensity-related effects on mechanistic changes in neurological and cardiovascular systems during stroke recovery. This thorough appraisal of HIIT and MICT cardiovascular exercise encompasses multiple domains pertaining to stroke rehabilitation and adherence to exercise (neuroplasticity, cardiovascular health, motor function, and psychosocial response to exercise).

Therefore, our findings will provide significant insight into the efficacy of multiple aspects of HIIT, in comparison to conventional MICT, as a new treatment modality for clinicians to employ safely and effectively for the benefit of individuals recovering from stroke. Findings from this study will inform clinicians involved with stroke rehabilitation and inform prescription of training programs to provide long-term benefit for health and functional recovery. Information obtained from this study will help in the individualized tailoring of exercise programs following stroke and provide integral support for future investigations into the application of HIIT in stroke populations, such as aspects of exercise dosage and type of training

## Trial status

This trial is currently ongoing, with activity and recruitment beginning in September 2019. After a hiatus in research activities due to COVID-19 shutdowns at both sites in March 2020, recruitment resumed in August 2020 and study activities are currently underway. It is expected that recruitment will be completed by April 2023.

## Supplementary Information

**Additional file 1.** Populated SPIRIT Checklist.

## Data Availability

Anonymized datasets that are prepared and/or analyzed during the current study will be available in the repository of journals upon publication. We will state in the published articles that data are available upon reasonable request to PIs MR and AT. These two investigators will have access to the final trial dataset.

## Abbreviations

HIIT: High-intensity interval training
MICT: Moderate-intensity continuous training
TMS: Transcranial magnetic stimulation
M1: Primary motor cortex
BDNF: Brain-derived neurotrophic factor
BP: Blood pressure
MRI/CT: Magnetic resonance imaging/computed tomography
JRH: Jewish Rehabilitation Hospital
HR: Heart rate
RPE: Rate of perceived exertion
HRR: Heart rate reserve
MoCA: Montreal Cognitive Assessment
MEP: Motor-evoked potential
RMT: Resting motor threshold
FDI: First dorsal interosseus muscle
CSE: Corticospinal excitability
SICI: Short intracortical inhibition
ICF: Intracortical facilitation
PWV: Pulse wave velocity
CPET: Cardiopulmonary exercise test
ECG: Electrocardiogram
10mWT: 10-m walk test
6MWT: 6-min walk test
PACES: Physical Activity Enjoyment Scale
BREQ-3: Behavioural Regulation in Exercise Questionnaire-3
